# Harmane Potentiates Nicotine Reinforcement Through MAO-A Inhibition at the Dose Related to Cigarette Smoking

**DOI:** 10.3389/fnmol.2022.925272

**Published:** 2022-06-27

**Authors:** Zheng Ding, Xiangyu Li, Huan Chen, Hongwei Hou, Qingyuan Hu

**Affiliations:** ^1^China National Tobacco Quality Supervision & Test Center, Zhengzhou, China; ^2^Key Laboratory of Tobacco Biological Effects, Zhengzhou, China; ^3^Joint Laboratory of Translational Neurobiology, Zhengzhou, China

**Keywords:** harmane, nicotine, monoamine oxidase (MAO), dopamine, cigarette smoking, substance abuse

## Abstract

Nicotine is the primary addictive component in cigarette smoke, and dopamine release induced by nicotine is considered a significant cause of persistent smoking and nicotine dependence. However, the effects of nicotine replacement therapy on smoking cessation were less effective than expected, suggesting that other non-nicotine constituents may potentiate the reinforcing effects of nicotine. Harmane is a potent, selective monoamine oxidase A (MAO-A) inhibitor found in cigarette smoke, but showed no effect on nicotine self-administration in previous studies, possibly due to the surprisingly high doses used. In the present study, we found that harmane potentiated nicotine self-administration on the fixed ration schedule at the dose related to human cigarette smoking by the synergistic effects in up-regulating genes in addiction-related pathways, and the effect was reduced at doses 10 times higher or lower than the smoking-related dose. The smoking-related dose of harmane also enhanced the increase of locomotor activity induced by nicotine, accompanied by increased dopamine basal level and dopamine release in the nucleus accumbens through MAO-A inhibition. Our findings provided new evidence for the important role of non-nicotine ingredients of tobacco products in smoking addiction.

## Introduction

According to the Federal Trade Commission Cigarette Report, cigarette sales went up for the first time in 20 years (Ma et al., 2022). High cigarette smoking rates are attributed to nicotine, the main component of tobacco products (Benowitz, 1992, 2010). However, nicotine is only one of more than 8,000 chemical constituents in tobacco products (Rodgman and Perfetti, 2008). Nicotine replacement therapy is not enough to relieve withdrawal symptoms in smokers (Rose et al., 2000). Other non-nicotine components in tobacco products contribute to smoking addiction, but underlying mechanisms are unclear (Rose, 2006). Nicotine stimulates dopamine release by activating dopamine neurons, which induces the rewarding effects (Rice and Cragg, 2004; De Biasi and Dani, 2011). Monoamine oxidase is responsible for dopamine metabolism, and brain monoamine oxidase activity in smokers is significantly lower than that of non-smokers (Fowler et al., 1996a,b). Inhibition of monoamine oxidase increases nicotine-induced dopamine release and reinforces the rewarding effects of nicotine (Meyer et al., 2006; Lewis et al., 2007).

MAO has two isoenzymes: MAO-A and MAO-B (Weyler et al., 1990). Although MAO-A and MAO-B have been shown to metabolize dopamine *in vitro*, MAO-B is absent in dopaminergic neurons and does not affect baseline dopamine levels, whereas MAO-A is primarily responsible for dopamine metabolism in the brain (Butcher et al., 1990; Cho et al., 2021). Previous work focused only on the effects of medicinal or clinical MAO inhibitors (Guillem et al., 2005, 2006; Villégier et al., 2006; Smith et al., 2016). Little evidence is available about the potentiating effect of MAO inhibitors in tobacco products. Among the monoamine oxidase inhibitory components in cigarette smoke components, two potent inhibitors were found in cigarette smoke to affect monoamine oxidase, harmane and norharmane (Poindexter and Carpenter, 1962; Ho et al., 1968; Rommelspacher et al., 2002; Herraiz and Chaparro, 2005). Norharmane was shown to enhance low-dose nicotine self-administration possibly through the MAO-A inhibition (Arnold et al., 2014). The inhibitory ability of harmane to MAO-A is about 20 times that of norharmane (Herraiz and Chaparro, 2005). During short-term smoking cessation in smokers, the plasma levels of harmane instead of norharmane were significantly correlated with the brain MAO-A activity (Bacher et al., 2011). Therefore, it was speculated that harmane might also potentiate the reinforcing effects of nicotine, but harmane did not affect nicotine self-administration in previous studies. However, it is worth noting that harmane was used at doses much higher than those found in tobacco smoke (equivalent to smoking 100–5000 cigarettes a day) and was pre-injected rather than co-administered with nicotine in these studies (Villégier et al., 2006; Hall et al., 2014). On the one hand, the activity of neurons in the nucleus accumbens were inhibited by excessive doses of harmane (Ergene and Schoener, 1993). On the other hand, the monoamine oxidase inhibition of harmane might resulted in the loss of the potentiation of nicotine reinforcing effect (Smith et al., 2016). New findings might have emerged if harmane doses were designed according to human smoking levels.

In addition to the MAO-A inhibition, harmane has various other properties, including psychoactive and pharmacological effects (Adell and Myers, 1994; Aricioglu and Altunbas, 2003; Aricioglu and Utkan, 2003; Aricioglu-Kartal et al., 2003; Khan et al., 2017). Since the potential effects of harmane may enhance or reducenicotine reinforcement, we performed an RNA-seq study to explore the synergistic effects of harmane and nicotine in regulating the expression of addiction related genes.

Therefore, this study focused on the potentiation mechanism of harmane on the reinforcing effects of nicotine. Firstly, the effect of harmane on nicotine reinforcement was investigated. Secondly, the effects of harmane and nicotine on the basal levels and dopamine release were determined. Thirdly, the potentiation mechanisms of harmane on the reinforcing effects of nicotine were investigated. Finally, the potential synergistic effects of harmane and nicotine in regulating the expression of addiction-related genes were explored. Our results demonstrate that at the dose related to human smoking, harmane potentiates the reinforcing effects of nicotine through MAO-A inhibition and the synergistic effects of harmane and nicotine up-regulated genes in addiction-related pathways.

## Materials and Methods

### Animals

Male Sprague Dawley rats (7 weeks, 200–250 g) were purchased from Beijing Vital River Laboratory Animal Technology Co., Ltd. When arrived in the laboratory, rats were placed in temperature, humidity, and pressure-controlled cages (light on from 8:00 p.m. to 8:00 a.m., test during dark time) and acclimated for a week before experiments. Rats were housed individually in cages with steel lids (for food storage) and filter tops and allowed free access to adequate water and food before the experiment. After starting the experiment, the rats were given ∼10 g (2∼3 pills) of food per day to keep weight. The experimental procedures were conducted according to the National Institutes of Health Guide for the Care and Use of Laboratory Animals. The procedures were approved by the Laboratory Animal Management and Ethics Committee of China National Tobacco Quality Supervision and Test Center (Approval Number: CTQTC-SYXK-2021002).

### Drugs

Nicotine bi-L-(+)-tartrate dihydrate was purchased from TCI (N0080). Harmane was purchased from MCE (HY-101392). Drugs were dissolved in 0.9% sterile saline (pH 7.0–7.4). Since harmane cannot be directly dissolved in 0.9% sterile saline, harmane was pre-dissolved with tartaric acid (TCI, T0025) solution consistent with nicotine bi-L-(+)-tartrate dihydrate. The drug solution was prepared freshly and stored at 4°C immediately after use. The doses of harmane in this study were selected according to the ratio of harmane and nicotine in the cigarette smoke based on the human daily smoking levels. Moreover, the harmane in our research was self-administered with nicotine rather than pre-injected in our study to simulate the situation of human smoking.

### Self-Administration Procedures

Before the experiment, the surgical instruments were sterilized with 70% ethanol and irradiated under ultraviolet light for 30 min. Rats were anesthetized with isoflurane and injected intramuscularly with Zoletil™ 50 (Virbac). An electric shaver was used to remove hair from the right chest and part of the back of the rat. The pulsating jugular vein was found in the rat’s right chest, and a pre-sterilized silicone tube was inserted. After the silicone tube is fixed, connect the other end of the silicone tube to the self-administration button (Instech, VABR1B/22) on the back. Silicone tubes were ventilated with saline containing heparin sodium and penicillin per day after the surgery. Rats were allowed to recover for at least 7 days before self-administration.

Self-administration program started without food training since food training enhances self-administration in rats (Valentine et al., 1997; Donny et al., 1998; Clemens et al., 2010; Garcia et al., 2014). Fixed ratio (FR) self-administration programs were used in this study (Weiss, 2013), where the number following FR represents the number of responses required to obtain a single drug injection. In FR1, FR2, and FR3 schedule, 1, 2, and 3 responses must be produced in order to obtain injections (Cooper et al., 2007). Rats were placed in the operant chambers for 1 h with FR1, FR2, FR3 self-administration schedule on days 1–4, 5–12, and 13–27, allowing rats to learn longer at higher FR. When an infusion is triggered, the chamber’s light will be turned off for 20 s, accompanied by a beeping sound for 1 s. During the light-off period, the nosepoke is invalid but still recorded.

### Open-Field Experiments

A total of 24 rats (6 in each of 4 groups) were used for subcutaneous injection, including 14 day saline (S), 14 day 10 μg/kg harmane (H), 14 day 300 μg/kg nicotine (N), and 14 day 300 μg/kg nicotine + 10 μg/kg harmane (N + H) (equivalent to the harmane smoked by a smoker with 20 cigarettes/day). Rats were given 10 ± 0.5 g of food per day to reduce the effects of the diet and weight. Open field experiments refer to the method in JOVE (Seibenhener and Wooten, 2015). For the locomotor activity test, rats were free to move around in the open field for 10 min. The central area of the open field was defined as 60 cm × 60 cm. The total distance represented the locomotor activity and correlated with nucleus accumbens dopamine release in rats. Before testing, rats were adapted to the testing room for 30 min. Rats were placed in the open field before the first injection and 5 min after the last injection. The background was snapshotted before the experiment as a reference value.

### MAO-A Level, MAO-A Activity, and Dopamine Level Determination

Rats were anesthetized with Zoletil 1 h after the last injection (refer to blood harmane return to baseline levels within 1 h after smoking a cigarette) and decapitated. The rat brains were washed and taken out with PBS in an ice-water mixture. Nucleus accumbens was extracted referred to the method of Jacobs et al. (2004). Pre-sterilized 0.5 mm iron bead was added to each ep tube and shook 20 times at 60 Hz for 30 s by Tissuelyser (Shanghai Jingxin, JXFSTPRP).

The homogenized samples were first centrifuged at 2000 rpm for 20 min, and 200 μL of the supernatant was taken to determine MAO-A activity and MAO-A level. The remaining samples were centrifuged at 5,000 rpm for 10 min (according to the recommended centrifugation speed of kits). The supernatants were filtered with a 0.22 μm membrane before determination. MAO-A activity was determined by the MAO-Glo™ Assay Systems kit (Promega, v1402). 10 nM (1.7 ng/mL) rasagiline in DMSO (Shanghai yuanye, S24846) was added to each well to reduce the influence of MAO-B. MAO-A and dopamine levels were detected using the Rat MAO-A ELISA kit (Laibio, JL39022) and Rat Dopamine ELISA kit (Laibio, JL12965). Fluorescence and luminescence were measured using the multimode microplate reader (Spark^®^, TECAN).

### Microdialysis and Dopamine Detection

A total of 17 rats were used for microdialysis after subcutaneous injection, 10 μg/kg harmane (*n* = 4), 300 μg/kg nicotine (*n* = 7), and 300 μg/kg nicotine + 10 μg/kg harmane (*n* = 6) (equivalent to the nicotine and harmane smoked by a smoker with 20 cigarettes/day). Rats were anesthetized with isoflurane and injected intramuscularly with Zoletil™ 50 (Virbac). An electric shaver was used to remove hair on the top of the head. Microdialysis probes and cannulas (CMA/12, cat No. 8010432) were sterilized with UV light before use. Anesthetized rats were head-fixed on the stereotaxic apparatus (RWD, 68025). According to the rat brain stereotaxic reference book (Paxinos and Watson, 2006), the position of the nucleus accumbens shell was set as AP: +2.5 mm, ML: +1.4 mm, and DV: -7.0 mm. The microdialysis experiment was performed 3–5 days after the recovery of the rat after the cannula implantation surgery. The rats were kept for 2 h after connecting to the microdialysis device to keep the baseline stable. The flow rate of microdialysis was set at 2.5 μl per minute, and samples were collected every 10 min. Each rat was measured with 10 time points, the first 4 points were the baseline values, and the drug was injected from the 4th point. The detection of dopamine uses high-performance liquid chromatography combined with an electrochemical detector (HPLC-ECD).

### RNA-Seq Study

Rats were anesthetized, decapitated, and brains removed within 2 h of the last self-administration. The nucleus accumbens of the rat was taken out for RNA-seq. After taking out the rat brain, blood and impurities were washed out with pre-chilled PBS. Nucleus accumbens was taken using a brain mold in an ice-water mixture and were immediately put into a liquid nitrogen box for storage. The samples were sent to Beijing Genomics Institute (BGI), where the RNA extraction and library construction were performed. High-throughput sequencing was completed using the BGISEQ-500 sequencing platform (Drmanac et al., 2010).

### Statistical Analysis

Data analysis was mainly based on ANOVA of repeated measures, multiple comparisons, and *t*-tests when appropriate through Graph pad prism 8.0. Data were represented in the figure as the mean ± SEM. The RNA-seq data were analyzed using the online analysis system Dr. Tom provided by BGI,^1^ and the visualization tool provided by Dr. Tom includes the drawing of bubble diagrams and Venn diagrams. Significance levels were set as *p < 0.05, ^**^p < 0.01, ^***^p < 0.001 and the screening conditions for differential genes were set as | log2FoldChange| > 0.5 and *Q-*value < 0.05.

## Results

### Harmane Potentiated Nicotine Self-Administration at the Dose Related to Human Smoking

**FIGURE 1:**
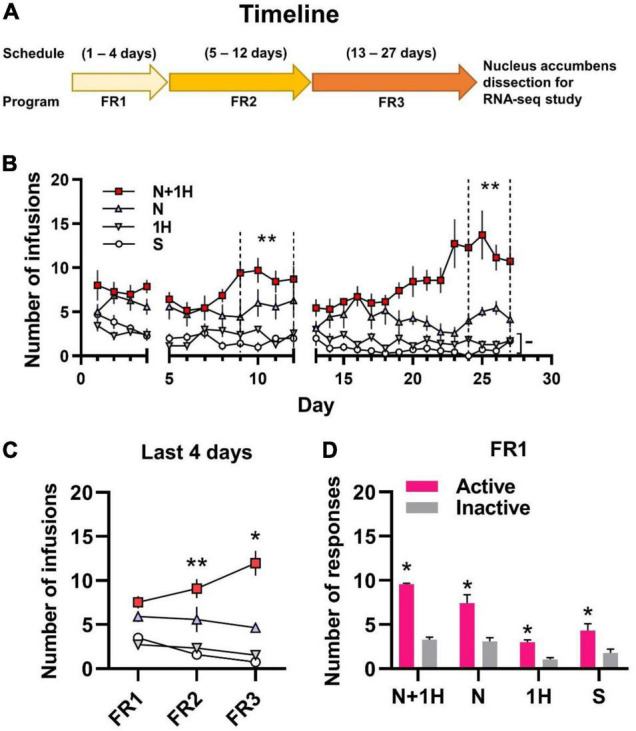
Self-administration of smoking-related doses of nicotine and harmane in rats. **(A)** Timeline of 1 h/day self-administration. **(B)** Number of infusions in FR1-FR3 self-administration. White circle, saline group (S); gray inverted triangle, 1 μg/kg/inf harmane group (1H); blue triangle, 30 μg/kg/inf nicotine group (N); red square, 30 μg/kg/inf nicotine + 1 μg/kg/inf harmane group (N + 1H). The number of infusions in FR2-FR3 was significantly increased in the N + 1H group compared to the N group (**p < 0.01), while there was no significant difference from FR1 to FR3 in the H and S groups (^–^*p* > 0.05). **(C)** Average infusions in the FR1-FR3 last 4 days in each group. The number of infusions increased significantly from FR1 to FR3 in the N + 1 group (**p < 0.01 from FR1 to FR2 and *p < 0.05 from FR2 to FR3), while other groups did not increase. **(D)** Histograms of the response data in FR1. Active responses were significantly higher in all groups than inactive responses (*p < 0.05). Data are presented in the figure using mean ± SEM.

A total of 49 rats (7 in each of 7 groups) were used for self-administration. In 3R4F cigarette smoke, the content ratio of harmane and nicotine is about 1:30 (Jaccard et al., 2019; van der Toorn et al., 2019). Therefore, groups in the rat self-administration experiment were set as: Saline (S), Nicotine (30 μg/kg/inj) (N), Harmane (1 μg/kg/inj) (1H), Nicotine (30 μg/kg/inj) + Harmane (1 μg/kg/inj) (N + 1H). The timeline of self-administration in rats is shown in Figure 1A, starting with FR1 for 4 days, followed by conversion to FR2 for 8 days, and finally FR3 for 15 days. The doses administered in each group of rats remained the same from FR1 to FR3, thus requiring more responses in FR2 and FR3 to maintain drug intake. FR1 was to allow rats learn self-administration (Chen et al., 2007), and FR2 to FR3 were used to investigate the maintenance and reinforcement of drugs (Sorge et al., 2009; De La Peña et al., 2015).

Rats in the nicotine group (N) actively self-administered nicotine compared to the S group in the last 4 days of FR2 and FR3 (*p < 0.05) but not in FR1 (^–^*p* = 0.0725). Rats in the N group maintained stable self-administration (*p* > 0.05 between FR1, FR2, and FR3) (Figures 1B,C). Rats in the harmane + nicotine group (N + 1H) showed active self-administration in the last 4 days of each FR (*p < 0.05) compared to the S group and FR2-FR3 to those of N group (^–^*p* = 0.1142, ^**^p = 0.0048 and ^**^p = 0.0023 for FR1-FR3) (Figures 1B,C). Comparing the last 4 days of each FR, harmane + nicotine further potentiated the number of infusions from FR1 to FR3 (FR2 vs. FR1 ^**^p = 0.0181 and FR3 vs. FR2 *p = 0.0343) (Figure 1C). No significant difference (^–^*p* = 0.7465, 0.6168, and 0.4508) was found between the S and H groups (*p* > 0.05) in the last 4 days of FR1-FR3 (Figures 1B,C). Rats in all groups learned to self-administer (*p* < 0.05 between active and inactive nosepokes) through the nature of nosepoke without food training in the FR1 program (Figure 1D). The results indicated that harmane potentiated nicotine self-administration but showed no reinforcing effects alone.

Reduced Potentiating Effect of Harmane on Nicotine Self-administration at Doses Not Related to Human Smoking.

**FIGURE 2:**
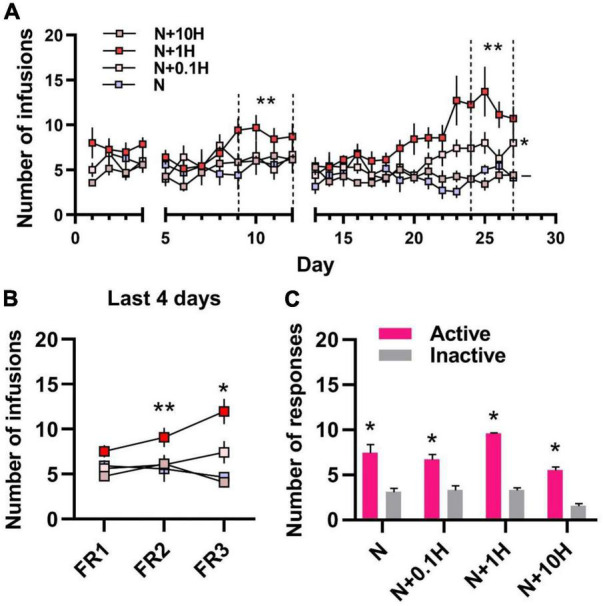
Self-administration of nicotine with different doses of harmane in rats. **(A)** Number of infusions in FR1-FR3 self-administration. Blue square, 30 μg/kg/inf nicotine group (N); pink square 30 μg/kg/inf nicotine + harmane (0.1 μg/kg/inf, N + 0.1H), red square, nicotine + harmane (1 μg/kg/inf, N + 1H) and brown square, nicotine + harmane (10 μg/kg/inf, N + 10H). The number of infusions in FR2-FR3 was significantly increased in the N + 1H group compared to the N group (**p < 0.01), while the N + 0.1H group was only significantly higher in FR3 (*p < 0.05), and the N + 10H group had no significant difference compared to the N group (^–^*p* > 0.05). **(B)** Infusions in the FR1-FR3 last 4 days in each group. The number of infusions increased significantly from FR1 to FR3 in the N + 1 group (**p < 0.01 from FR1 to FR2 and *p < 0.05 from FR2 to FR3), while other groups did not increase. **(C)** Histograms of the response data in FR1. Active responses were significantly higher in all groups than inactive responses (*p < 0.05). Data are presented in the figure using mean ± SEM.

To further examine the effects of harmane at different doses on nicotine reinforcement, nicotine (30 μg/kg/inj) + harmane (10 μg/kg/inj) (N + 10H) and nicotine (30 μg/kg/inj) + harmane (0.1 μg/kg/inj) (N + 0.1H) were also designed. The timeline of self-administration in rats was referred to the timeline in Figure 1A without RNA-seq study. As expected, no significant potentiating effects were shown in harmane at 10 times doses on nicotine self-administration in each FR (^–^*p* = 0.4378, 0.7720, and 0.7423) (Figures 2A,B). Nicotine self-administration were not affected by 0.1 μg/kg/inj harmane in the last 4 days of FR1 and FR2 (^–^*p* = 0.9946 and 0.9531), but a significant increase was found in the last 4 days of FR3 (*p = 0.0150) compared to those of N group (Figures 2A,B). The reinforcing effect of harmane at 1 μg/kg/inj (N + 1H) was significantly higher than at 10 and 0.1 μg/kg/inj in the last 4 days of FR3 [one-way ANOVA, *F*_(3.000, 7.544)_ = 66.00, N + 1H vs. N + 10H ^**^p = 0.0018 and N + 1H vs. N + 0.1H ^**^p = 0.0085] (Figure 2B). A main effect of harmane doses was revealed by 3 × 4 two-way ANOVA [*F*_(2.174, 13.04)_ = 15.42, *p* = 0.0003] but not FR [*F*_(1.834, 11.00)_ = 1.636, *p* = 0.2383], and no significant interaction between harmane dose and FR [F_(2.299, 13.79)_ = 2.493, *p* = 0.1138] (Figure 2C). Both N + 10H and N + 0.1H groups learned to self-administer in FR1 (*p* < 0.05 between active and inactive nosepokes) (Figure 2D). The results indicated that harmane potentiated nicotine self-administration within a range of doses and the potentiating effect was reduced at doses beyond this dose range. The results of the number of responses were consistent with the results of the number of infusions (Supplementary Figure 3).

### Harmane Increased Basal Levels of Dopamine by Inhibiting MAO-A and Synergized With Nicotine to Potentiate Locomotor Activity in Rats

**FIGURE 3:**
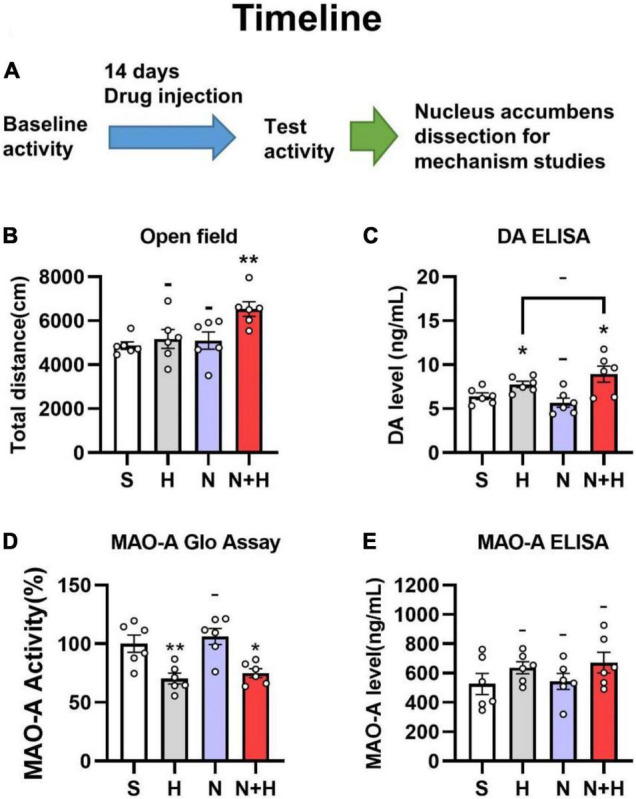
**(A)** Timeline of subcutaneous administration in rats. **(B)** Effects of nicotine and harmane on the open field total distance of the rats in S, H, N, and N + H groups after drug injection. Rats in the N + H group significantly increased the open field total distance (**p < 0.01), while no significant difference were found in the other groups. **(C)** Effects of nicotine and harmane on the basal level of dopamine in the nucleus accumbens. Compared to the S group, the dopamine level in the H and N + H groups increased significantly (*p < 0.05), but there was no significant change in the N group. **(D)** Effects of nicotine and harmane on the activity of MAO-A in the nucleus accumbens (NAc) of rats. Compared to the S group, the MAO-A activity in the H and N + H groups decreased significantly (*p < 0.05), but there was no significant change in the N group. **(E)** Effects of nicotine and harmane on levels of MAO-A in the nucleus accumbens (NAc) of rats. No significant difference was found in the all four groups. Data are presented in the figure using mean ± SEM.

One of the striking differences between nicotine and other addictive drugs concerns its locomotor effects. Unlike other addictive drugs enhance locomotor activity, nicotine showed weak or no effect in animals (Villégier et al., 2006). Unlike other addictive drugs enhance locomotor activity, nicotine showed weak or no effect in animals (Marks et al., 1983; Freeman et al., 1987; Whiteaker et al., 1995), but the synergistic effects of nicotine with MAO inhibitors resulted in increased locomotor activity (Villégier et al., 2003, 2006). Since harmane is a potential monoamine oxidase inhibitor, we used open field tests to examine whether the synergistic effects of harmane and nicotine cause locomotor activity changes in rats. The injection dose of nicotine and harmane was set at 300 μg/kg/day and 10 μg/kg/day (30:1) (Figure 3A), equivalent to the nicotine and harmane intake through 20 cigarettes by a smoker a day (Matta et al., 2007).

Rats in each group showed similar locomotor activity before subcutaneous injection of the drug (Supplementary Figure 1). A significantly increased locomotor activity were shown in the harmane and nicotine (N + H) groups than in the saline (S) group (^**^p = 0.0025) after 14 days of co-administration (Figure 3B). Harmane and nicotine alone did not induce significant change in locomotor activity (*p* > 0.05 compared to the S group). These findings indicated that the synergistic effects of harmane and nicotine rather than alone caused the increase in locomotor activity.

MAO-A inhibition proved to increase basal levels of dopamine by inhibiting dopamine metabolism, which increases dopamine release upon stimulation of neuronal signaling (Cho et al., 2021). Although harmane is a MAO-A inhibitor *in vitro* studies (Herraiz and Chaparro, 2005), whether harmane can cause MAO-A inhibition and increase basal dopamine levels have not been investigated. Therefore, immunoassays (ELISA kits for dopamine determination) were used to examine whether harmane potentiates nicotine reinforcement by increasing basal dopamine levels. Since the nucleus accumbens is the main brain area for dopamine release, metabolism and recycling in the drug reward pathway (Di Chiara et al., 2004), the samples used were the nucleus accumbens of rats that completed the open-field experiments. A significant increase in the dopamine basal level of in the nucleus accumbens was found in the H and N + H groups (*p = 0.0240 and 0.0402) (Figure 3C). Nicotine alone or in combination with harmane did not significantly affect dopamine levels (*p = 0.2868 and 0.2771) (Figure 3C). Elevated baseline dopamine levels induced by harmane provided a basis for harmane to increase nicotine-induced dopamine release (Cho et al., 2021).

In addition to examining basal dopamine levels, we asked whether these changes are induced by harmane-induced MAO-A inhibition. The inhibition of MAO-A is not only related to the inhibition of MAO-A activity but also associated with the expression levels of MAO-A also contribute to MAO-A activity. Therefore, the effects of nicotine and harmane on MAO-A in the nucleus accumbens were examined. The samples used to determine MAO-A levels, and MAO-A activity was the nucleus accumbens of rats that completed the open field experiment. Instead of causing changes in MAO-A levels in nucleus accumbens (Figure 3E), harmane inhibited MAO-A activity (^**^p = 0.0078) (Figure 3D). Nicotine alone did not cause changes in MAO-A activity (^–^*p* > 0.05), nor did it synergize with harmane (^–^*p* > 0.05 compared H group to the NH group) (Figure 3D).

### Harmane Potentiated and Prolonged Nicotine-Induced Dopamine Release

**FIGURE 4:**
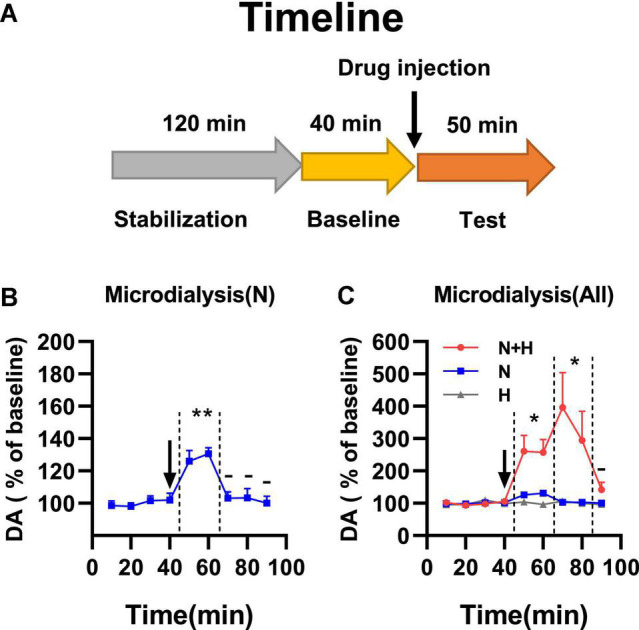
**(A)** Timeline of microdialysis study. **(B)** Effects of nicotine alone on dopamine release in nucleus accumbens. Significant dopamine release was showed in N group within 20 min of drug injection (**p < 0.01). **(C)** Effects of harmane and nicotine on dopamine release in nucleus accumbens. N + H group showed significantly increased release of dopamine compared to N group (*p < 0.05) in the first 20 min, and maintained high levels of extracellular dopamine in 40 min after injection compared to N group (*p < 0.05). Data are presented in the figure using mean ± SEM.

Smoking-related doses of harmane induced MAO-A inhibition in nucleus accumbens and increased dopamine basal levels. To verify whether harmane increased nicotine-induced dopamine release, a microdialysis study was performed (Figure 4A). The injection dose of nicotine and harmane was set at 300 μg/kg and 10 μg/kg (consistent with behavioral experiments). Nicotine-induced dopamine release within 20 min of drug injection (^**^p = 0.0014), and dopamine returned to basal levels after 30 min (^–^*p* > 0.05 compared to basal levels) (Figure 4B). The synergistic effects of harmane with nicotine increased the release of dopamine (*p = 0.0263 compared to N group) in the first 20 min, and maintained high levels of extracellular dopamine in 40 min after injection (*p = 0.0375 comparing the N + H group with the N group) and returned to basal levels after 50 min (Figure 4C). No significant effects were found on baseline extracellular dopamine levels of the H group (^–^*p* > 0.05 compared to baseline, not shown in the figure). Results indicated that harmane may potentiate nicotine reinforcement through a mechanism of increasing the dopamine release induced by nicotine.

### The Synergistic Effects of Harmane and Nicotine Increased the Number of Genes Affected in Addiction-Related Pathways

In addition to being an MAO-A inhibitor, harmane has other psychoactive and pharmacological effects that may contribute to nicotine reinforcement. To further reveal these potential effects, transcriptomic analysis of the nucleus accumbens of rats in the S, 1H, N, and N + 1H groups were performed by RNA sequencing (RNA-Seq) after self-administration studies. The N + 1H group was chosen because it has a higher potentiating effect than the N + 0.1H and N + 10H groups. Considering that 1H group hardly self-administered harmane, we injected the rats in group H with the dose of harmane equivalent to the N + H group in the last 4 days of FR2 (10 inf × 1 μg/kg/inf = 10 μg/kg) to hold the 30:1 ration. Injecting harmane did not alter harmane infusions (^–^*p* = 0.1152) between the last 4 days of FR2 and FR3 of the 1H group. Nucleus accumbens samples from the 4 groups of rats were renamed SAL, HAR, NIC, and NH, such that SAL_NAC_1 refers to the nucleus accumbens of the first rat in the saline group.

**FIGURE 5:**
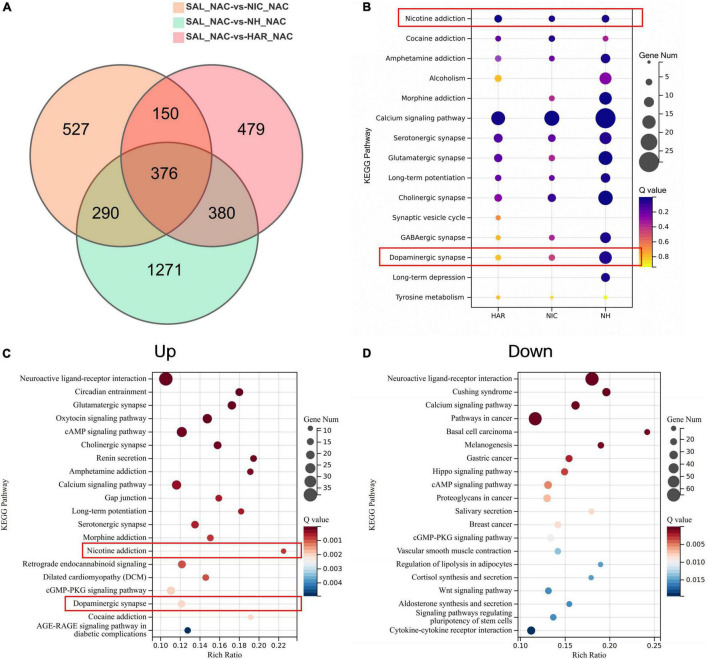
Analysis of differential genes and pathways in the nucleus accumbens transcriptome of self-administered rats. **(A)** Venn diagram of differential genes in three-group alignment. SAL, HAR, NAC, and NH refer to the S, 1H, N, and N + 1H groups. NAC refers to the nucleus accumbens. **(B)** Bubble plot of addiction-related pathways enriched for differential genes in the NH group. HAR, NIC, and NH refer to the intersection of HAR and NH (290 + 376), the intersection of NIC and NH (380 + 376), and the non-intersection of NH (1271). Bubble plot of pathways enriched by up-regulated **(C)** and down-regulated **(D)** genes in nucleus accumbens of NH group.

The transcriptomes of the HAR, NIC, and NH groups were compared with those of the S group, and the resulting differentially expressed genes (DEGs) (| log2FC| > 0.5 and *Q*-value < 0.05) were represented by Venn diagrams (Figure 5A. We focused on the DEGs in NH groups, as the synergistic effect of harmane and nicotine showed the strongest self-administration. For the DEGs in the NH group, 290 + 376 DEGs were induced by the administration of nicotine (section named NIC), 380 + 376 DEGs were induced by the administration of harmane (section named HAR), and the other 1271 DEGs were induced by the synergistic effects of harmane and nicotine (section named NH).

Next, the Kyoto Encyclopedia of Genes and Genomes (KEGG) pathway enrichment analysis was performed according to the genes of each section (NIC, HAR, and NH sections) and found that addiction-related pathways were significantly enriched (Figure 5B). Not only the nicotine reinforcement and the dopaminergic neuron pathways have a significant response under the combined action of harmane and nicotine, but also the addiction pathways of other drug addiction (cocaine, morphine, amphetamine addiction, and alcoholism) and various other neurotransmitters (serotonin, glutamate, GABA, and choline) pathways all exhibit the responses that harmane and nicotine alone do not. In addition, the synergistic effect of harmane and nicotine also produced responses related to memory in the calcium signaling pathway, long-term potentiation, and long-term depression. It can be seen from the bubble chart responses induced by harmane and nicotine alone contributed only part of the effect, and more response genes were observed only in the co-administration of harmane and nicotine (Figure 5B).

To better understand the synergistic effects of harmane and nicotine on the nucleus accumbens, KEGG pathway enrichment analysis was performed for the DEGs that were up-regulated (Up) and down-regulated (Down) in the NH group (Figures 5C,D). We were surprised to find that the genes of the nicotine addiction and dopaminergic pathways were significantly up-regulated in the top 20 most significant pathways. The activation of the addiction-related pathways facilitated the reinforcement of self-administration and locomotor behaviors in rats. In addition, possibly due to increased dopamine release, the dopamine D2 receptor gene Drd2 was up-regulated, and the tyrosine hydroxylase gene Th was down-regulated (Supplementary Figure 2 and Supplementary Table 1), which might lead to a block in dopamine synthesis (El Mestikawy et al., 1986; Lindgren et al., 2001), and these effects might reflect the loss of potentiation at high doses of harmane. The genes of the top 20 most significant pathways downregulated by the synergistic effects of harmane and nicotine were not directly associated with addiction but were primarily associated with cancer and disease, reflecting the pharmacological functions of harmane and nicotine. Similar to the findings in self-administration and dopamine release, the synergistic effects of harmane and nicotine produced profound responses in the nucleus accumbens, including nicotine reinforcement and the dopaminergic pathways. Taken together, it can be speculated that with the increase of the dopamine release induced by the synergistic effects of harmane and nicotine, the addiction-related pathways are activated, and self-administration is potentiated (Dayan, 2009; Subramaniyan and Dani, 2015).

## Discussion

Our study demonstrated that, at the dose related to human cigarette smoking, (1) nicotine self-administration was potentiated by harmane and reduced at 10× or 0.1× doses; (2) locomotor activity was increased by the synergistic effects of nicotine and harmane; (3) dopamine basal levels and nicotine-induced dopamine release were increased by harmane through MAO-A inhibition; (4) the pathways related to nicotine reinforcement were reinforced by the synergistic effects of nicotine and harmane. These findings suggest that harmane, a non-nicotine component in tobacco products, plays a role in smoking addiction through MAO-A inhibition.

It is worth noting that appropriate doses and approaches were performed compared to previous studies of harmane and other MAO inhibitors-using the dose related to human cigarette smoking and co-injection with nicotine. Excessive doses of harmane inhibited the activity of nucleus accumbens neurons in a previous study (Ergene and Schoener, 1993). The level of harmane to activate neurons in the nucleus accumbens is comparable to the level achieved by cigarette smoke (Talhout et al., 2007). Our study suggests that there exists a dose range of harmane to show strengthening effects. Previous studies used harmane beyond this dose range, where harmane exhibited no effects. Similar to an earlier study of acetaldehyde strengthening nicotine self-administration, the three doses used in the study were only twofold different, causing acetaldehyde to lose its reinforcement. Interestingly, harmane is related to acetaldehyde since acetaldehyde reacts with tryptamine and 5-HT to produce harmane (Talhout et al., 2007). The intake of ethanol also increases harmane. The primary metabolite of ethanol is acetaldehyde (Collins, 1988), indicating that the conversion of ethanol and acetaldehyde into harmane may also be important factors to reinforce nicotine reinforcement.

The effect of MAO-A inhibition may not only directly inhibit MAO-A but can also affect the MAO-A through gene and protein alteration. Previous studies on harmane inhibition of MAO-A were conducted *in vitro*, which were inappropriate since another factor was overlooked - the level of MAO-A. This study demonstrated that subcutaneous injection of smoking-related doses of harmane inhibited rat brain MAO-A activity rather than affected the expressions. Therefore, we not only examined the inhibition of MAO-A by harmane but also examined the effect of harmane on MAO-A levels and proved that harmane only inhibited MAO-A but not the expression of MAO-A. Similar to the inhibition of human brain MAO-A by smoking, the inhibition of harmane on MAO-A at a dose related to smoking is also partial. With the assistance of harmane, nicotine-induced dopamine release increased, and thus nicotine reward was reinforced. Other drugs may become more addictive when combined with harmane as well, since inhibition of MAO-A and drugs involved in the dopaminergic reward pathway may be affected and become an addiction-susceptible state. Future research should provide greater insight into the properties of other substances in cigarette smoke that may significantly affect nicotine reinforcement, even if they are weak or non-addictive.

Interestingly, the combination of harmane and nicotine also affected other pathways. The pathways with neurotransmitters and other addictive substances are enhanced, possibly because harmane inhibiting MAO-A may cause changes in other neurotransmitters, as MAO-A metabolizes dopamine and participates in the metabolism of different neurotransmitters (Shih et al., 1999). Changes in these neurotransmitters may contribute to reinforcing addictive pathways, including nicotine. Glutamatergic (Tzschentke and Schmidt, 2003; Chen et al., 2021), cholinergic (Mansvelder et al., 2003), serotonergic (Dhonnchadha and Cunningham, 2008), and GABAergic (Xi and Stein, 2002) are potentially linked to drug addiction, revealing a reinforcing pathway for co-use of smoking and other addictive drugs. Harmane may inhibit addiction to these substances at high doses, as we found that harmane inhibit the expression of tyrosine hydroxylase (Th). Genes in some disease- and cancer-related pathways are down-regulated, which suggests pharmacological effects of nicotine and harmane.

There are several limitations exist in our studies. Firstly, we do not have a positive control for MAO-A inhibition, as these substances may affect nicotine addiction through other mechanisms and are not present in tobacco products (Lotfipour et al., 2011; Villégier et al., 2011). There are technical barriers to stable regulation of MAO-A activity by gene knockout and knock-in and may affect normal activities in rats (Bortolato et al., 2013; Singh et al., 2013). Secondly, due to the difficulty of *in vivo* detection of MAO-A and basal dopamine levels, these indicators are all detected *in vitro* and cannot reflect the changes of each rat before and after drug injection. Thirdly, the doses we used were based on 3R4F cigarette smoke, which might be different in other tobacco products. Fourthly, although subcutaneous nicotine injection is a common and convenient method of administration, the rate of nicotine uptake is relatively rapid compared with smoking. However, when nicotine is administered by injection, peak brain nicotine levels are significantly lower than those achieved by intravenous injection or smoking (Turner, 1975; Benowitz and Iii, 1984), so higher doses of nicotine were used in rats due to differences in metabolic rates between humans and rats (Gorrod and Jenner, 1975; Clarke and Kumar, 1983; Shoaib et al., 1994; Hukkanen et al., 2005; Matta et al., 2007). Finally, our study demonstrates the potentiation of harmane at the smoking dose, but revealing a maximum effect is time consuming. Despite these limitations, our findings underscore the importance of examining potential addiction-potentiating substances in tobacco products.

In conclusion, this study demonstrated that harmane, a monoamine oxidase inhibitor of tobacco smoke components, potentiated nicotine self-administration at human smoking-related doses. The involving mechanism of harmane is MAO-A inhibition which increases dopamine level nicotine-induced dopamine release. The nicotine pathway was strengthened by the synergistic effects of harmane and nicotine, which provided evidence of pathway changes contributing to nicotine reinforcement. Taken together, other non-nicotinic ingredients in tobacco products and their underlying mechanisms remain a need for further research, which helps to reveal the mechanism of smoking addiction and the factors that contribute to smoking.

## Data Availability Statement

Publicly available datasets were analyzed in this study. This data can be found here: https://ngdc.cncb.ac.cn/gsub/ and the accession number is PRJCA009953 (https://ngdc.cncb.ac.cn/search/?dbId=&q=%20PRJCA009953).

## Ethics Statement

The animal study was reviewed and approved by the Laboratory Animal Management and Ethics Committee of China National Tobacco Quality Supervision and Test Center (Approval Number: CTQTC-SYXK-2021002).

## Author Contributions

ZD, XL, HC, HH, and QH conceived and designed the experiments. ZD, XL, and HC performed the experiments. ZD performed the data analyses and drafted the manuscript writing. ZD and XL revised the manuscript. All authors have read and approved the manuscript.

## Conflict of Interest

The authors declare that the research was conducted in the absence of any commercial or financial relationships that could be construed as a potential conflict of interest.

## Publisher’s Note

All claims expressed in this article are solely those of the authors and do not necessarily represent those of their affiliated organizations, or those of the publisher, the editors and the reviewers. Any product that may be evaluated in this article, or claim that may be made by its manufacturer, is not guaranteed or endorsed by the publisher.

## Footnotes

1
https://biosys.bgi.com/
