# Supplementary material for: Harmane Potentiates Nicotine Reinforcement Through MAO-A Inhibition at the Dose Related to Cigarette Smoking
Source: Front Mol Neurosci. 2022 Jun 27;15:925272. doi: 10.3389/fnmol.2022.925272 (PMC9271706; doi:10.3389/fnmol.2022.925272)
Supplement: Supplementary file 1 [file Data_Sheet_1.docx]

Supplementary Material

## Supplementary Figures and Tables


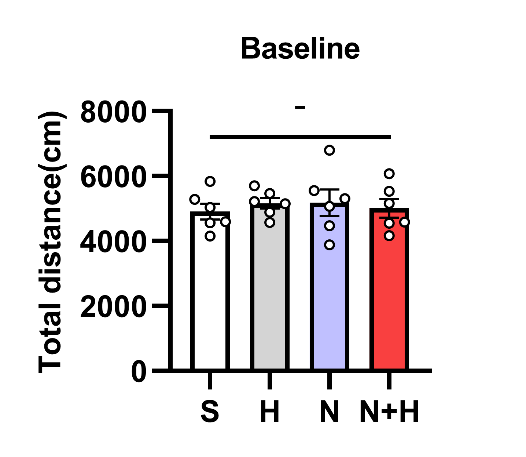


**Supplementary Figure 1.** Open field total distance of the rats in S, H, N, and N+H groups before drug injection.


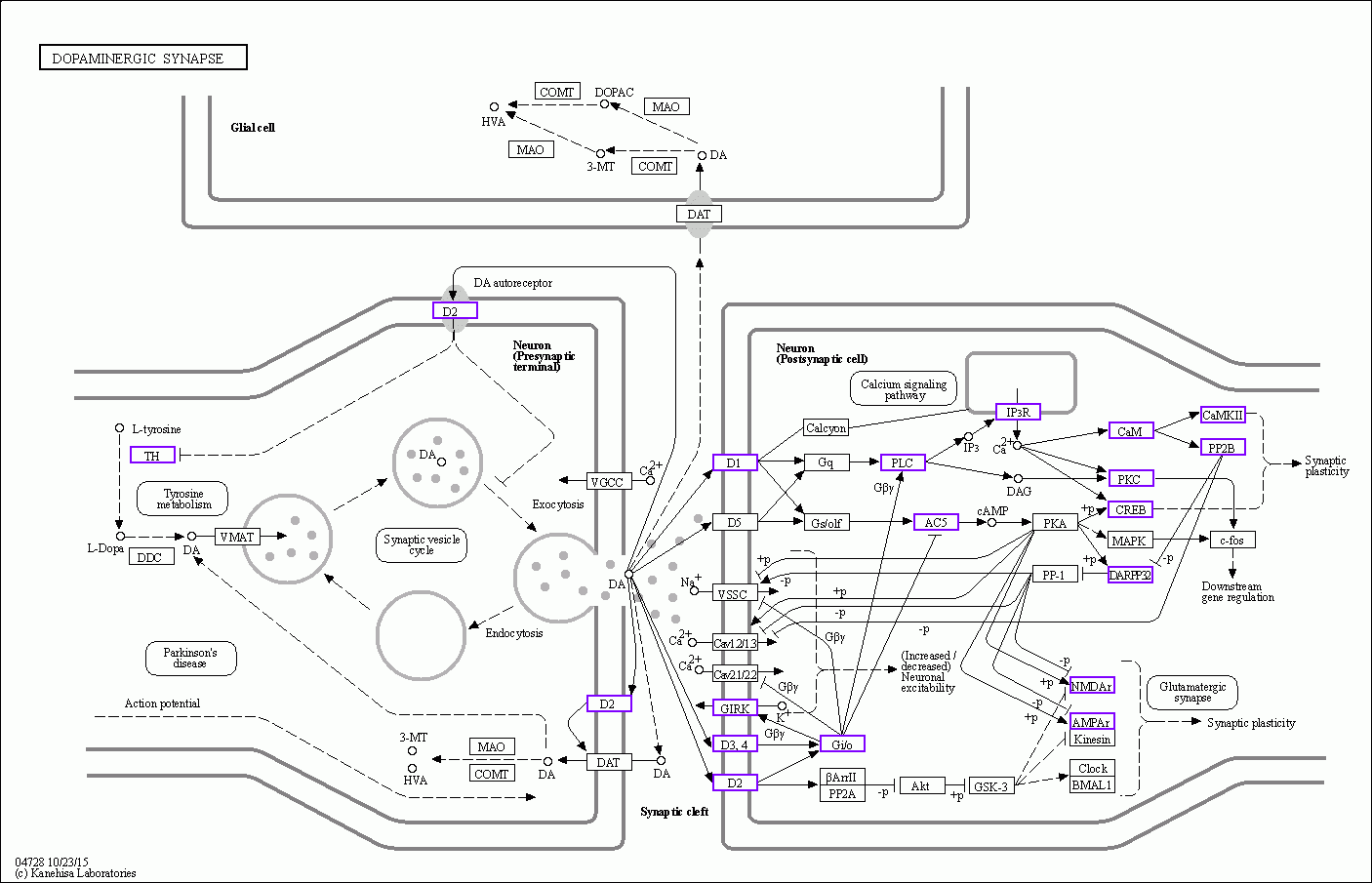


**Supplementary Figure 2.** Differentially expressed genes affected in dopaminergic neuron pathway

Table 1 Changes of differential genes in dopaminergic pathway

| Gene ID | Gene Symbol | log2 (HAR_NAC / SAL_NAC) | log2 (NIC_NAC / SAL_NAC) | log2 (NH_NAC / SAL_NAC) |
| --- | --- | --- | --- | --- |
| 103693838 | 'NEWGENE_621802' | -0.18 | 0.03 | -0.64 |
| 108349548 | 'LOC108349548' | 0.02 | 0.49 | -0.50 |
| 114120 | 'Gng12' | -0.21 | -0.07 | -0.55 |
| 24245 | 'Camk2b' | 0.21 | 0.19 | 0.55 |
| 24316 | 'Drd1' | 0.54 | 0.19 | 0.93 |
| 24318 | (D2)'Drd2' | 0.45 | 0.69 | 0.77 |
| 24410 | 'Grin2b' | 0.47 | 0.09 | 0.62 |
| 24654 | 'Plcb1' | 0.48 | 0.04 | 0.50 |
| 24674 | 'Ppp3ca' | 0.54 | 0.44 | 0.90 |
| 25023 | 'Prkcb' | 0.44 | 0.32 | 0.54 |
| 25031 | 'Plcb4' | -0.59 | -0.85 | -0.68 |
| 25085 | (TH)'Th' | -0.51 | -0.51 | -0.62 |
| 25262 | 'Itpr1' | 0.46 | 0.66 | 0.65 |
| 29238 | 'Drd3' | 0.10 | 0.22 | 0.75 |
| 294962 | 'Gnb4' | 0.15 | -0.01 | 0.54 |
| 29627 | 'Gria2' | 0.48 | 0.03 | 0.72 |
| 29628 | 'Gria3' | 0.46 | 0.08 | 0.62 |
| 29713 | 'Kcnj5' | -0.12 | 0.04 | -0.58 |
| 360616 | 'Ppp1r1b' | 0.33 | 0.67 | 0.65 |
| 362165 | 'Creb3l1' | -0.66 | 0.20 | -0.80 |
| 500131 | 'Creb5' | -0.22 | -0.24 | -0.64 |
| 60449 | 'Gnb3' | -0.25 | 0.50 | 0.92 |
| 64532 | 'Adcy5' | 0.39 | 0.59 | 0.60 |
| 685513 | 'Gng14' | 0.28 | -0.01 | 0.80 |
| 691455 | 'Calml4' | -0.17 | 0.02 | -0.99 |

**Supplementary Table 1.** Differentially expressed genes affected in dopaminergic neurons

**Supplementary Figure 3** Number of responses in FR1-FR3 self-administration. Blue square, 30μg/kg/inf nicotine group (N); pink square 30μg/kg/inf nicotine + harmane (0.1μg/kg/inf, N + 0.1H), red square, nicotine + harmane (1μg/kg/inf, N + 1H) and brown square, nicotine + harmane (10μg/kg/inf, N + 10H). The number of responses in FR2-FR3 was significantly increased in the N+1H group compared to the N group (^***^*p* < 0.001), while the N+0.1H group was only significantly higher in FR3 (^**^*p* < 0.01), and the N+10H group had no significant difference compared to the N group (^–^*p* > 0.05)
